# Risk factors and trajectories of opioid use following total knee replacement

**DOI:** 10.1186/s43019-022-00148-0

**Published:** 2022-04-05

**Authors:** Ralph Ward, David Taber, Haley Gonzales, Mulugeta Gebregziabher, William Basco, Jenna McCauley, Patrick Mauldin, Sarah Ball

**Affiliations:** 1 Department of Surgery, Medical University of South Carolina, 96 Jonathon Lucas Street CSB 426, Charleston, SC 29425 USA; 2 Department of Public Health Sciences, Medical University of South Carolina, Charleston, SC USA; 3 Department of Pediatrics, Medical University of South Carolina, Charleston, SC USA; 4 Department of Psychiatry and Behavioral Science, MUSC, Charleston, SC USA; 5 Department of Medicine, The Medical University of South Carolina, Charleston, SC USA

**Keywords:** Total knee arthroplasties (TKA), Pain, Opioids, Chronic opioid use (COU)

## Abstract

**Background:**

Opioids are commonly used to manage orthopedic pain in those undergoing total knee arthroplasty (TKA). There are limited studies assessing patterns of perioperative opioid use and risk factors for chronic use in patients undergoing TKA.

**Methods:**

This is a retrospective longitudinal cohort study of Medicaid enrollees undergoing TKA between 2014 and 2017 using de-identified medical and pharmacy claims. The primary outcome was chronic opioid use (opioid prescription filled 90–270 days following TKA). Trajectory group membership was determined by identifying distinct groups of patients with similar patterns of daily morphine milligram equivalent (MME) values during the postsurgery follow-up period.

**Results:**

In total, 1666 TKA surgeries performed in 1507 patients were included; 69% of patients were classified as chronic opioid users. Multivariable analyses identified prior opioid use, high opioid doses during the month after TKA, concomitant mood therapies and benzodiazepines, and comorbid conditions as important risk factors. Group-based trajectory analysis identified five distinct post-TKA surgery opioid use phenotypes with several key characteristics predicting group membership.

**Conclusions:**

This large-scale analysis demonstrated that chronic opioid use was common after TKA surgery and established several important risk factors for chronic use following TKA. Novel analysis revealed five distinct opioid use trajectories and identified key characteristics to help guide clinicians when determining perioperative opioid use. Results demonstrate that interventional studies attempting to reduce opioids after TKA are needed if reductions in long-term use are to be realized in this high-risk patient population.

**Supplementary Information:**

The online version contains supplementary material available at 10.1186/s43019-022-00148-0.

## Background

Osteoarthritis of the knee (OA) is a common chronic musculoskeletal condition. The progression of OA is characterized by a gradual increase in pain and a decrease in functionality and quality of life. OA management starts with pharmacotherapy and physical therapy to relieve pain and increase mobility. Pharmacological pain management typically begins with nonsteroidal antiinflammatory drugs (NSAIDs), followed by opioids for more severe pain [1]. Because severe OA requiring intense pain management is usually referred for surgical consideration, a significant number of patients undergoing orthopedic consultation are actively taking opioids [2]. Orthopedic surgery is a well-documented driver of chronic opioid prescription [9], and orthopedic surgeons rank third among opioid-prescribing specialties [2]. Alarmingly, in recent years, prescribing of opioids to manage OA has increased by 40% [2].

Total knee arthroplasty (TKA) is recognized as one of the most painful orthopedic procedures; 60% of patients who undergo TKA experience severe postoperative pain [3, 4]. Postoperative pain inhibits early ambulation, delays initiation of physical therapy, and prolongs the rehabilitation process [5, 6]. Although conventional pain management strategies rely heavily upon oral or intravenous opioid therapy, this approach is associated with risks. Undesirable side effects, including nausea, constipation, and somnolence, may delay ambulation [5]. Importantly, opioid use carries the risk of developing tolerance and dependence [6]. Following TKA, it is expected that patients experience a decline in pain and a decrease in analgesic requirements; unfortunately, chronic opioid use is reported in more than one-third of those undergoing TKA [7].

In response to the current opioid crisis, there is an urgent need to focus on perioperative interventions to reduce overall opioid use in TKA. [3, 8, 9]. Studies have suggested that cyclooxygenase-2 (COX-2) inhibitors, other non-opioid analgesics, and peripheral nerve blockade are effective at reducing postoperative opioid use [3, 6, 8–10], but more research is needed in this area. Given the commonality of chronic opioid use in patients undergoing TKA, it is important to have more information on trends in perioperative opioid use and risk factors for prolonged use, and to identify safe and effective approaches to perioperative pain management. For the analysis presented here, we hypothesized that chronic opioid use was common in a high-risk adult Medicaid population undergoing TKA and that there were significant risk factors associated with chronic opioid use and trajectories of use following this surgery, both of which can provide useful guidance to clinicians caring for these patients.

## Methods

### Study design and population

This was a retrospective longitudinal cohort study aimed at assessing utilization patterns and risk factors for chronic opioid use following TKA based on methods we described previously in assessing opioid use following pediatric tonsillectomy [11]. The institutional review board provided exempt review for this investigation. We obtained de-identified medical and pharmacy claims data for surgeries performed on Medicaid enrollees in South Carolina between January 2014 and December 2017 from the state’s Revenue and Fiscal Affairs Office (RFA). We also obtained Medicare part-D data from RFA for those patients with dual coverage. Surgeries were identified through appropriate ICD-9-CM and ICD-10-CM procedure codes, and are provided in Additional file 1: Table S1.

**Fig. 1:**
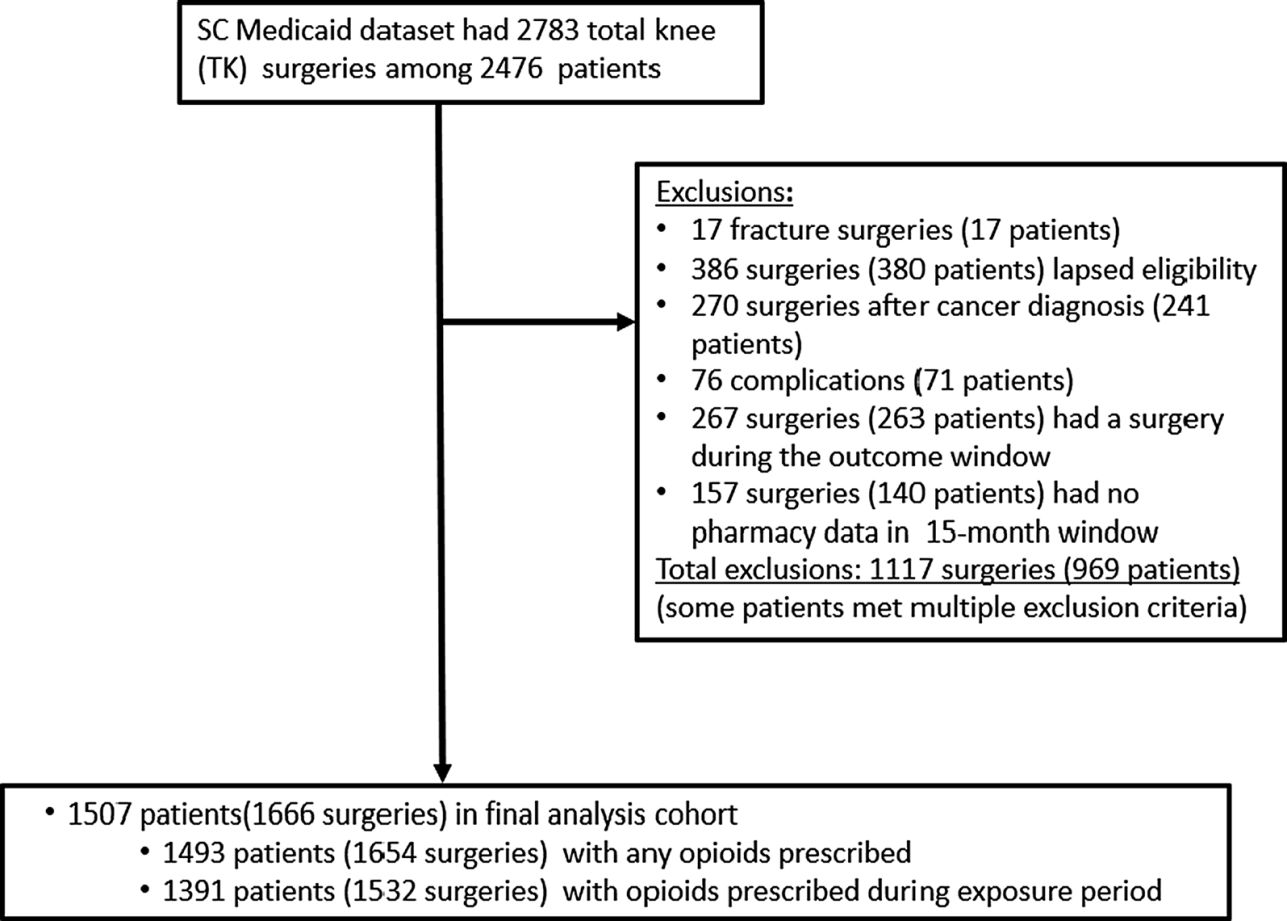
Patient screening flow chart

**Fig. 2:**
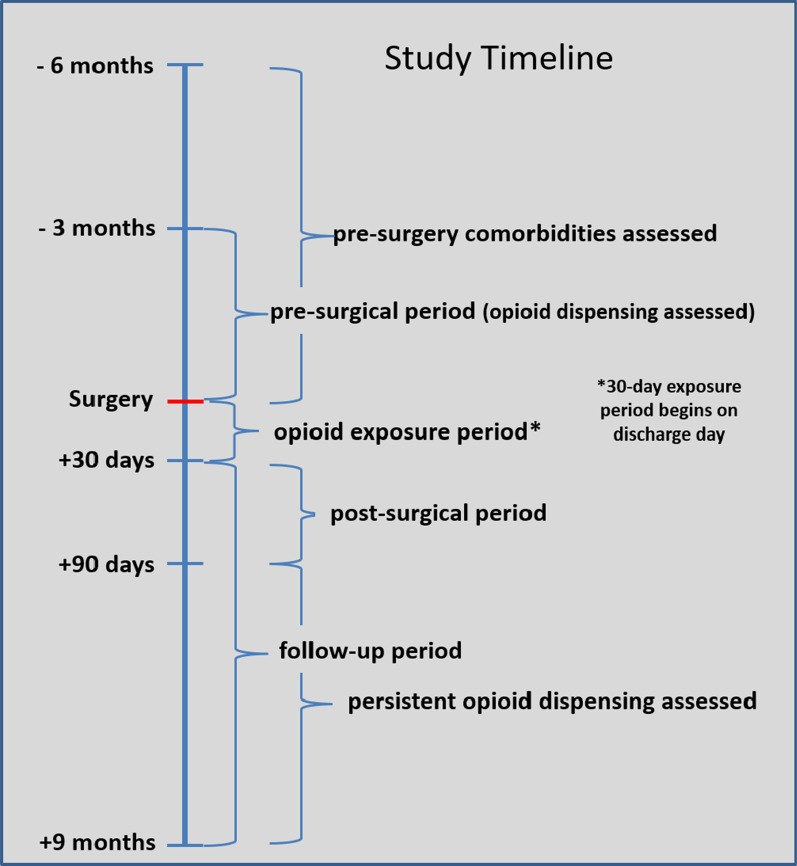
Study timeline

Figure 1 displays the number of surgeries and patients in the SC Medicaid dataset meeting criteria, as well as the final cohort of patients. The unit of analysis was individual surgeries (see exclusions following). Patients were excluded if any presurgery ICD code was found related to cancer, where we combined definitions described by Quan [12] and Feudtner [13]. Patients were also excluded if the diagnosis codes associated with the surgery indicated it was performed following an accident involving bone fractures (i.e., traumatic injury). Patients were followed from 6 months prior to the surgery date until 9 months post-discharge for the index TKA surgery. Patients without continuous Medicaid coverage throughout the 15-month period were excluded. To associate opioid use patterns with a single surgery, patients were excluded if they had a second knee surgery or another significant surgery during the 9-month post-discharge period that likely involved the use of opioids. Therefore, a patient could appear twice in the analyses and contribute to different outcome groups if their surgeries were > 9 months from discharge of the other TKA. This determination was based on a review of associated procedure codes. Patients were also excluded if their pharmacy data were missing during the pre- or postsurgery follow periods. Opioid use was determined through prescribing records. Opioid dose was calculated by converting opioid medications into morphine milligram equivalent (MME) daily dose using conversion factors provided by the US Centers for Disease Control and Prevention [14]. Dispensed opioids that were in liquid or patch form were excluded in 58 of 1666 surgeries owing to anomalies in the Medicare Part D data. In all but one surgery, the patient received other opioids in pill form. However, sensitivity analyses were performed in which models were compared that included or excluded liquids and patches, and no significant differences between models were found. We identified baseline comorbidities from ICD codes recorded during the 6-month presurgery period using Elixhauser definitions validated by Quan, supplemented by osteoporosis and sleep apnea, as these have been associated with opioid consumption [12]. Figure 2 displays the study timeline, where day 0 is the surgery discharge date, the presurgery period was 90 days prior to surgery, and the exposure period was the 30 days following discharge. Days 31–270 comprised the outcome follow-up period. The unit of analysis was individual surgeries.

### Outcomes

The primary outcome was chronic opioid use, defined as any opioid prescription filled ≥ 90 days to 270 days following discharge. A secondary outcome was trajectory group membership based on patient clusters with similar trajectories of opioid use during the follow-up period, where opioid use was assessed as monthly averages of daily morphine milligram equivalent (MME) values.

### Exposure and covariate definitions

We accounted for the following demographic variables in our statistical models: age (on date of surgery), sex, race–ethnicity group (non-Hispanic white, non-Hispanic Black, Hispanic, and other/unknown group). Opioid use was determined for the presurgery, exposure, and follow-up periods using mean days per month with dispensed opioids, mean MME over 30 days (MME per day), and the proportion of patients with dispensed opioids. We also accounted for the following baseline opioid variables during the 90-day presurgery period: opioid naïve was defined as 0 opioid days; daily opioid use was categorized as 0–49, 50–89, and ≥ 90 MME per day, on the basis of CDC-defined cut points. Exposure period variables included the number of opioid prescriptions during the 30-day period, the opioid type(s) (single versus combination, long-acting versus short-acting medications), the providers’ practice specialties, other medications co-prescribed with opioids (antipsychotics, antidepressants, gabapentin and pregabalin, selected anxiolytics, muscle relaxants, benzodiazepines, non-benzodiazepine sedative/hypnotics, duloxetine). Variables related to the surgery and process of care included length of stay, treatment at a skilled nursing or rehabilitation facility, rehospitalization within 30 days, number of days from discharge until the first visit with a surgeon or primary care provider, total number of visits to each provider type, and overall total visits.

### Statistical analyses

As described in detail in a previous publication [11] bivariate analyses were performed using *t*-tests, ANOVA, chi-squared tests, or Fisher’s exact tests, as appropriate. We estimated risk ratio (RR) and 95% confidence interval (CI) for all measured risk factors using generalized linear mixed models (GLMM) with a Poisson distribution, and a log link [15]. We estimated relative risk instead of odds ratios because the chronic outcome was relatively common in this population [16]. We used two models to assess the effects of opioid exposure (days of opioid exposure or mean MME per day during the 30-day exposure period). We also used group-based trajectory models (GBTM) to identify clusters of surgical procedures where patients had similar patterns of MME per day during the 8-month follow-up period. These models are based on finite mixture model methods, with an assumed zero-inflated Poisson distribution for the MME responses [17–19]. We used SAS PROC TRAJ and considered models with up to fourth-order polynomials and up to six trajectory groups. The best model in terms of numbers of groups was selected on the basis of the Bayesian information criterion (BIC). Parameter estimates for the highest-order polynomial with *p* < 0.05 were used to determine the best-fitting curve for each group. Finally, we used a multinomial logistic model to predict which variables were most influential in predicting group assignment. A subanalysis was conducted assessing the etiologies of ED visits and hospitalizations during the follow-up period, assessed using ICD 9/10 codes and AHRQ Clinical Classification Software (CCS) [20]. All analyses were performed using SAS 9.4 (SAS Institute, Inc., Cary, NC).

## Results

From 2014 to 2017, there were 1666 TKA surgeries performed in 1507 patients (Table 1) meeting eligibility criteria. Among all procedures, 78.9% involved same-day surgeries or one-night hospital stays, and 69.9% of patients undergoing TKA were classified as chronic opioid users, defined as opioid use 90–270 days after discharge. When comparing chronic versus non-chronic opioid use groups, there were significantly fewer chronic users with no opioid use in the 90 days prior to surgery (19.3% versus 59.6% respectively, *p* < 0.0001), and opioids were prescribed to patients with chronic use at a substantially higher rate during the exposure period (94.3% versus 69.1% respectively, *p* < 0.0001). Chronic opioid users were younger (mean age 58.7 versus 62.0 years, *p* < 0.0001), more likely to be co-prescribed concomitant medications including antidepressants (30.2% versus 18.0%, *p* < 0.02), benzodiazepines (19.0% versus 7.7%, *p* < 0.0001), and muscle relaxants (14.0% versus 4.8%, *p* < 0.0001), while being more likely to visit additional prescribers during the 1-month postsurgery period (mean opioid prescribers: 1.7 versus 1.0, *p* < 0.0001). Chronic opioid users had a higher mean number of chronic comorbidities (6.7 versus 6.0, *p* < 0.0001), particularly pulmonary disease, liver disease, substance use disorders, and rheumatoid arthritis (Table 2).

**Table 1:** Demographic and clinical characteristics for total knee replacement, SC Medicaid 2014–2017

Measure^a^	Level	Chronic^b^	Non-chronic	Total	*p*-Value
Unique patients^c^		1054 (69.9%)	500 (30.1%)	1507	
Number of total knee surgeries	First surgery	1050 (69.7%)	457 (30.3%)	1507	0.059
	Second surgery	99 (62.3%)	60 (37.7%)	159	
Age, years	Mean [standard deviation (sd)]	58.7 (9.8)	62.0 (10.1)	59.7 (10.0)	< 0.0001
Sex	% male	233 (20.3%)	98 (19.0%)	331 (19.9%	0.53
Race/ethnicity	White	420 (36.6%)	176 (34.0%)	596 (35.8%)	0.044
	Black	517 (45.0%)	223 (43.1%)	740 (44.4%)	
	Hispanic	6 (0.5%)	9 (1.7%)	15 (0.9%)	
Baseline measures (presurgery period^d^)					
Opioid naïve^e^	0 days	222 (19.3%)	308 (59.6%)	530 (31.8%)	< 0.0001
Opioid use during presurgery period	0–49 MME/day	999 (87.0%)	509 (98.5%)	1508 (90.5%)	< 0.0001
	50–89 MME/day	112 (9.8%)	8 (1.6%)	120 (7.2%)	
	≥ 90 MME/day	38 (3.3%%)	0 (0%)	38 (2.3%)	
Opioid use^f^ among all patients					
Mean opioid days, mean (std)	Presurgery period	44.0 (42.0)	12.7 (10.6)	34.2 (35.0)	< 0.0001
	Exposure period^g^	20.0 (19.5)	10.5 (9.7)	17.1 (10.4)	< 0.0001
	≥ 90 days postsurgery	85.4 (81.7)	0	58.9 (66.9)	< 0.0001
Mean MME, mean(std)	Presurgery period	21.8 (32.6)	5.9 (12.8)	17.6 (30.0)	< 0.0001
	Exposure period	58.8 (55.6)	27.5 (25.0)	49.1 (51.2)	< 0.0001
	≥ 90 days postsurgery	25.1 (41.2)	0	18.5 (42.7)	< 0.0001
Opioid prescriptions dispensed during exposure period					
Mean opioid days	Mean (std)	21.2 (20.7)	15.2 (14.4)	19.7 (8.5)	< 0.0001
Opioid prescription count	Mean (std)	3.1 (1.5)	2.2 (1.3)	2.8 (1.5)	< 0.0001
Provider practice specialty	Surgeon	769 (66.9%)	274 (53.0%)	1043 (62.6%)	< 0.0001
	Primary care provider	173 (15.1%)	50 (9.7%)	223 (13.4%)	
Concomitant medications dispensed during exposure period (all patients)					
	NSAIDS, APAP	263 (22.9%)	99 (19.2%)	362 (21.7%)	0.089
	Antidepressants	347 (30.2%)	93 (18.0%)	440 (26.5%)	< 0.02
	Gabapentin, pregabalin	229 (19.9%)	51 (9.8%)	280 (16.8%)	< 0.02
	Benzodiazepines	218 (19.0%)	40 (7.7%)	258 (15.5%)	< 0.0001
	Selected sedatives/hypnotics^h^	108 (9.4%)	22 (4.3%)	130 (7.8%	0.0002
	Muscle relaxants	161 (14.0%)	25 (4.8%)	186 (11.2%)	< 0.0001
Surgery and process of care variables					
Rehospitalization, *N* (%)	Within 30 days of discharge	21 (1.8%)	5 (1.0%)	26 (1.2%)	0.28
Hospital length of stay	2 or more nights	254 (22.1%)	97 (18.8%)	351 (21.1%)	0.13
	1 night or same day	895 (77.9%)	420 (81.2%)	1315 (78.9%)	
Number of unique providers visited (claims data)	Mean (std)	1.17 (1.09)	1.00 (0.88)	1.21 (1.43)	0.02
Count of distinct opioid prescribers (pharmacy data)	Mean (std)	1.7 (0.85)	1.0 (0.87)	1.46 (0.92)	< 0.0001

*APAP* acetaminophen, *MME* morphine milligram equivalent, *NSAIDS* nonsteroidal antiinflammatory drugs

^a^Because some patients had multiple surgeries, unless otherwise indicated, all measures are aggregated by surgical procedures rather than by individual patients

^b^Chronic use was defined as any opioid prescription filled ≥ 90 to 270 days after discharge from procedure

^c^Sum of patients with chronic and non-chronic use does not reflect value in total column because some patients with multiple surgeries may appear in both the chronic and non-chronic groups

^d^The presurgery period was defined as the 90 days prior to procedure

^e^Opioid naïve for analyses defined as zero days of opioid use during the presurgery period

^f^Opioid use was assessed by dispensed opioid prescriptions

^g^The exposure period was defined as the 30 days immediately after discharge from procedure

^h^This category includes non-benzodiazepine sedative/hypnotics and selected anxiolytics

**Table 2:** Medical comorbidities by chronic opioid outcome *N*(%)

Condition	Chronic	Non-chronic	Total	*p*-Value
Congestive heart failure	203 (17.7%)	87 (16.8%)	290 (17.4%)	0.73
Valvular disease	175 (15.2%)	81 (15.7%)	256 (15.4%)	0.83
Pulmonary circulation disease	90 (7.8%)	27 (5.2%)	117 (7.0%)	0.062
Peripheral vascular disease	180 (15.7%)	68 (13.2%)	248 (14.9%)	0.21
Hypertension without complications	1029 (89.6%)	461 (89.2%)	1490 (89.4%)	0.8
Hypertension with complications	164 (14.3%)	57 (11.0%)	221 (13.3%)	0.073
Paralysis	38 (3.3%)	20 (3.9%)	58 (3.5%)	0.56
Other neurological disorders	233 (20.3%)	101 (19.5%)	334 (20.0%)	0.74
Chronic pulmonary disease	625 (54.4%)	218 (42.2%)	843 (50.6%)	< 0.0001
Diabetes without chronic complications	378 (32.9%)	191 (36.9%)	569 (34.2%)	0.12
Diabetes with chronic complications	288 (25.1%)	133 (25.7%)	421 (25.3%)	0.81
Hypothyroidism	223 (19.4%)	104 (20.1%)	327 (19.6%)	0.74
Renal failure	191 (16.6%)	73 (14.1%)	264 (15.8%)	0.22
Liver disease	153 (13.3%)	36 (7.0%)	189 (11.3%)	0.0001
Peptic ulcer disease	35 (3.0%)	12 (2.3%)	47 (2.8%)	0.53
Acquired immune deficiency syndrome	11 (1.0%)	8 (1.5%)	19 (1.1%)	0.32
Rheumatoid arthritis	262 (22.8%)	88 (17.0%)	350 (21.0%)	0.008
Coagulopathy	60 (5.2%)	18 (3.5%)	78 (4.7%)	0.13
Obesity	727 (63.3%)	310 (60.0%)	1037 (62.2%)	0.21
Weight loss	91 (7.9%)	37 (7.2%)	128 (7.7%)	0.62
Fluid and electrolyte disorders	399 (34.7%)	156 (30.2%)	555 (33.3%)	0.073
Chronic blood loss anemia	49 (4.3%)	24 (4.6%)	73 (4.4%)	0.7
Deficiency anemias	434 (37.8%)	195 (37.7%)	629 (37.8%)	1.0
Alcohol abuse	81 (7.0%)	30 (5.8%)	111 (6.7%)	0.4
Substance use disorder	195 (17.0%)	45 (8.7%)	240 (14.4%)	< 0.0001
Psychoses	244 (21.2%)	100 (19.3%)	344 (20.6%)	0.4
Osteoporosis	98 (8.5%)	45 (8.7%)	143 (8.6%)	0.92
Depression	507 (44.1%)	187 (36.2%)	694 (41.7%)	0.003
Opioid use disorder	100 (8.7%)	22 (4.3%)	122 (7.3%)	0.001
Sleep apnea	366 (31.9%)	163 (31.5%)	529 (31.8%)	0.91
Number of chronic conditions, mean (std)	6.7 (3.2)	6.0 (2.9)	6.5 (3.1)	< 0.0001

On multivariable analysis for the outcome of chronic opioid use, those with presurgical opioid use had 60% greater risk for chronic use (RR 1.60, 95% CI 1.45–1.78). Patients receiving 1–7 days, 8–29 days, or 30 days of opioids during the 1-month postsurgery period had an increasingly higher risk of 66% to 108% for chronic opioid use (RR 1.66, 95% CI 1.30–2.13; RR 1.88, 95% CI 1.51–2.35; RR 2.08, 95% CI 1.67–2.60, respectively; Table 3). Each additional opioid prescription filled during the 1-month postsurgery period was associated with 3% increased risk of chronic use (RR 1.03, 95% CI 1.01–1.05). Patients with longer hospital stays had 7% increased risk, compared with those with one night or less (RR 1.07, 95% CI 1.00–1.14). During the 1-month postsurgery period, prescribing of antipsychotics increased the risk of chronic opioid use by 14% (RR 1.14, 95% CI 1.03–1.26). Patients with several chronic comorbid conditions also had higher risk of chronic opioid use: pulmonary circulation disorders (RR 1.13, 95% CI 1.02–1.26) and liver disease (RR 1.09, 95% CI 1.01–1.17). A multivariable model that used mean MME per day during the 1-month postsurgery period as the primary exposure variable provided similar results (far column in Table 3). Of note, age was not a significant predictor in the adjusted models but was in the univariate associations.

**Table 3:** Regression analyses (GLMM) predicting chronic opioid use (opioids ≥ 90 to 270 days after total knee replacement)^a^

Variable	Level	Risk ratio (95% CI) for chronic opioid use	
		Days of opioid exposure	MME exposure
Opioid naïve^b^	No versus yes	1.60 (1.45, 1.78)	1.63 (1.47, 1.80)
Opioid days during exposure period^c^	1–7 days versus 0 days	1.66 (1.30, 2.13)	
	8–29 days versus 0 days	1.88 (1.51, 2.35)	
	30 days versus 0 days	2.08 (1.66, 2.60)	
Mean daily MME during exposure period	0 < MME < 50 versus 0 MME		1.80 (1.45, 2.24)
	50 ≤ MME < 90 versus 0 MME		1.91 (1.53, 2.40)
	MME ≥ 90 versus 0 MME		1.92 (1.52, 2.44)
Hospital length of stay	4+ days versus 3 or fewer days	1.07 (1.00, 1.15)	1.07 (1.00, 1.14)
Number of unique opioid prescribers during exposure period	Per additional prescriber	1.01 (0.97, 1.06)	1.02 (0.98, 1.06)
Patient age	Per additional year	1.00 (1.00, 1.00)	1.00 (0.99, 1.00)
Number of opioid prescriptions during exposure period	per additional prescription	1.03 (1.01, 1.05)	1.03 (1.01, 1.06)
Race/ethnicity	White	Ref.	Ref.
	Minority race–ethnicity	1.05 (0.99, 1.12)	1.05 (0.99, 1.12)
Sex	Female	Ref.	Ref.
	Male	0.98 (0.91, 1.06)	0.98 (0.91, 1.06)
Other medications during exposure period	NSAIDS, APAP	0.97 (0.91, 1.04)	0.97 (0.91, 1.04)
	Antidepressants	1.03 (0.97, 1.10)	1.04 (0.97, 1.11)
	Antipsychotics	1.14 (1.03, 1.26)	1.14 (1.02, 1.26)
	Gabapentin	1.05 (0.97, 1.12)	1.05 (0.98, 1.13)
	Pregabalin	1.02 (0.91, 1.16)	1.02 (0.91, 1.15)
	Benzodiazepines	1.01 (0.95, 1.08)	1.01 (0.95, 1.08)
	Selected sedatives/hypnotics^d^	1.05 (0.97, 1.13)	1.04 (0.97, 1.13)
	Muscle relaxants	1.07 (1.00, 1.15)	1.07 (1.00, 1.15)
	Duloxetine	0.98 (0.89, 1.09)	0.98 (0.88, 1.08)
Medical comorbidities	Pulmonary circulation disease	1.13 (1.02, 1.26)	1.13 (1.02, 1.26)
	Diabetes without complications	0.94 (0.88, 1.00)	0.93 (0.87, 1.00)
	Liver disease	1.09 (1.01, 1.17)	1.09 (1.01, 1.17)

*APAP* acetaminophen, *GLMM* generalized linear mixed modeling, *MME* morphine milligram equivalent, *NSAIDS* nonsteroidal antiinflammatory drugs, *95% CI* 95% confidence interval

^a^Chronic opioid use defined as any opioid prescription filled ≥ 90 to 270 days following discharge for the index TKA surgery

^b^Opioid naïve for analyses defined as 0 opioid days during the presurgery period

^c^The exposure period was defined as the 30 days immediately after discharge from procedure

^d^This category includes non-benzodiazepine sedative/hypnotics and selected anxiolytics

**Fig. 3:**
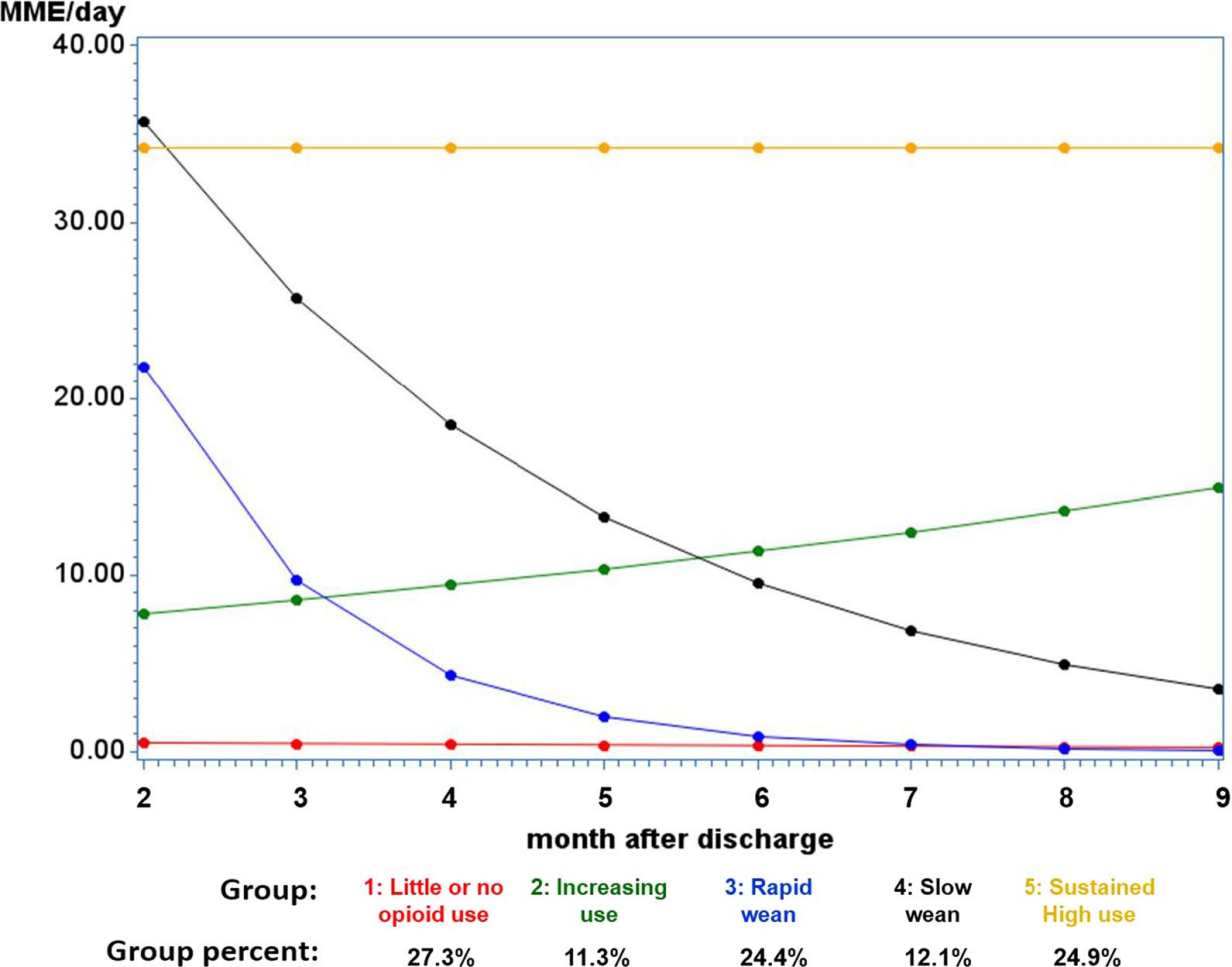
Opioid MME trajectory groups

Group-based trajectory analysis determined that post-exposure MME trajectory patterns were best explained by five distinct phenotypes (Fig. 3 and Additional file 1: Table S2). These included “little or no opioid use” (group 1, 27.3%), “increasing opioid use” (group 2, 11.3%), “rapid opioid wean” (group 3, 24.4%), “slow opioid wean” (group 4, 12.1%), and “sustained high opioid use” (group 5, 24.9%). Univariate analysis (Additional file 1: Table S2) demonstrated several characteristics that differed by group. Patients in groups 4 and 5 (“slow wean” and “sustained high opioid use”), versus group 1, had higher rates of chronic use, were younger, had high mean MME per day during pre- and postsurgery periods, had higher opioid prescription counts, were more likely to have concomitant medications prescribed with opioids, and had the highest number of unique opioid prescribers during the exposure period. Groups 2 and 3 (“increased opioid use” and “rapid wean”) were characterized by intermediate values in each risk predictor, including opioid naïve status, the fraction with pre- and postsurgery opioid consumption, number of exposure period prescription counts and opioid exposure days, the proportion with concurrent medication use, and number of unique opioid prescribers.

In multinomial models predicting group membership (Additional file 1: Table S3), group 5 membership (“sustained high use”) was associated with greater numbers of opioid prescriptions and substantially higher mean MME per day during the exposure period, concomitant use of benzodiazepines, antidepressants and muscle relaxants, and a longer hospital length of stay after surgery. This group was also more likely to have diagnoses for substance use disorder, diabetes with complications, and rheumatoid arthritis. Comparison of groups 3 and 4 (“rapid opioid wean” and “slow wean”) indicated that a rapid wean was associated with older age and with patients who were more likely to be opioid naïve and to have lower opioid exposure after the surgery. Comparison between groups 1 and 2 (“little or no opioids” versus “increasing opioid use”) indicated that increased use was associated with multiple opioid prescriptions, being non-opioid naïve, and having a history of substance use disorder, perivascular disease, and chronic blood loss anemia.

A subanalysis was conducted for the two groups with the most concerning trajectories (group 2, increasing opioid use, and group 5, sustained high opioid use) to further examine the role of postsurgery medical events in increasing or sustaining high opioid use during the outcome period. Specifically, etiologies of emergency department (ED) visits and hospitalizations (using primary ICD 9/10 codes and AHRQ CCR software) were analyzed and are displayed in Additional file 1: Table S4, S5 (group 2), S6, and S7 (group 5). The results demonstrate that the most common reasons for ED visits in the post-exposure period were cardiovascular, infections, acute injuries, and treatment of comorbidities for groups 2 and 5; however, opioid utilization did not appreciably change before, during, or after these visits. Common reasons for hospitalizations for groups 2 and 5 included infections, treatment of comorbid conditions, and TKA-related surgical complications. Opioid utilization did not appreciably change during or after hospitalization, regardless of etiology. Finally, in multivariate modeling, ED visits were predictive for group 5 membership (OR 2.13, 95% CI 1.56–2.90) but not group 2 (OR 1.41, 95% CI 0.974–2.04), as compared with group 1 membership. There were no significant associations between hospitalizations and group membership during the outcome period.

## Discussion

The results of this large-scale statewide longitudinal analysis demonstrate that chronic opioid use after TKA surgery is very common in this high-risk adult population of adult Medicaid enrollees; 69% of patients who underwent TKA filled a prescription for opioids 90–270 days after surgery. Multivariable analyses demonstrated several important risk factors, including prior opioid use, high prescribing during the 1 month after surgery, concomitant prescribing of mood medications and benzodiazepines, and several comorbidities. Novel trajectory analysis of longitudinal opioid use following TKA surgery categorized post-exposure period opioid use into five distinct phenotypes: low to no use, high sustained use, rapid wean, slow wean, and increasing use. Group membership was predicted by several key risk variables.

At 69%, the rate of chronic opioid use following TKA found in this study is significantly higher than in previous studies, commonly citing rates of 30–50% [7]. There are several likely reasons for this. First, this study focused on an adult Medicaid population, and it is well established that public health insurance is a proxy for low socioeconomic status (SES). Previous research demonstrates that low SES and Medicaid, in and of itself, are risk factors for high rates of opioid use and substance use disorders [21–24]. Further, the analysis presented here represents a “real-world” population, as previous opioid use and abuse were not criteria for exclusion. Thus, the high rate of chronic opioid use seen in this analysis is likely a reflection of focusing on a high-risk population and limiting exclusion criteria. We have consistently found, when analyzing this Medicaid population, higher than previously cited opioid use when we evaluated other surgeries, including appendicitis, cholecystectomy and colon resections (unpublished), and tonsillectomy [11].

Consistent with our findings, previous research has identified preoperative opioid use as one of the most important risk factors for prolonged opioid use following TKA [7, 25, 26]. One analysis including 243 patients who underwent TKA found that patients who reported current opioid use the day of surgery were 53% more likely to report persistent opioid use at 6 months versus opioid-naïve patients [22]. Our work supports these findings; those with presurgical opioid use were at 60% greater risk for chronic use. We also assessed opioid use trajectories during the postoperative period and found that patients with an increasing trajectory in the 8 months following surgery were substantially less likely to be opioid naïve at the time of surgery. These patients were more likely to have a history of substance use disorder, additional comorbidities, and multiple opioid prescriptions during the 30 days following discharge for the surgery. Thus, previous opioid use and abuse are clear and significant risk factors for presenting with a chronic opioid use phenotype after TKA surgery. Clearly, interventional efforts should be focused on these high-risk patients in an effort to mitigate long-term opioid use following TKA surgery.

Additional characteristics beyond preoperative opioid status impacted postoperative opioid use and weaning. Those with low opioid exposure (based on mean daily MMEs) during the 1 month following surgery were more likely to be in the rapid wean group. Thus, reducing postoperative opioid prescribing is key to minimizing the risk of developing chronic opioid use following TKA. To this end, there is a growing and promising body of literature to suggest periarticular multimodal drug injections (PMDI) and femoral nerve blocks may be effective at controlling pain and reducing opioid consumption following TKA surgery. In a meta-analysis of ten randomized controlled trials, which included 744 TKAs, Wang et al. demonstrated that single femoral nerve block injection may be more effective for early postoperative pain relief while continuous PMDI was more efficacious for overall postoperative pain control. However, the authors state there is high variability in outcomes using these pain control modalities, and future studies are warranted before hard conclusions can be drawn [27, 28]. This offers a promising area of future research.

The two most concerning groups were group 2 (increasing opioid use) and group 5 (sustained high opioid use). Given the limitations of claims data, it is unclear why patients were increasing or taking large amounts of opioids during the follow-up time. An assessment of causes of ED visits and hospitalizations that occurred during the follow-up period (Additional file 1: Table S4 through S7) provided limited information, as opioid use remained consistent during and after these encounters, regardless of visit etiology [20]. Future studies are warranted in these troubling groups, using granular chart review to assess the true—likely multifactorial—etiologies of high opioid use in these groups.

Strengths of this study include the longitudinal design and large sample size of a traditionally at-risk population. Previous studies in the TKA population were predominantly limited to single-center analyses including limited numbers of patients [29, 30]. This analysis also benefited from a comprehensive analysis of opioid prescribing using medical and pharmacy claims data from both Medicaid and Medicare Part D repositories. There are several limitations to this study. First, baseline comorbidities were identified through ICD codes [12, 13]. This method may have missed certain comorbidities and misclassified others. Another limitation is that we did not identify indications for preoperative opioid use and could not identify patients who may have been prescribed opioids to treat pain unrelated to osteoarthritis or TKA. Relative to secondary surgeries, we were unable to distinguish between primary and secondary TKA surgeries, which may impact opioid use patterns and trajectories. The use of SC Medicaid data limits the generalizability of these results to populations in other states or outside Medicaid enrollment [21, 22]. We were unable to distinguish between drugs taken on an “as needed” versus scheduled basis; thus, the most likely MME exposure per day was used to estimate MME calculations. We also could not capture hospital-administered opioids, so we cannot assess the relationship between inpatient opioid exposure and outcomes following discharge. Our access to only administrative data prevented us from assessing true causes of opioid prescribing, and it is important to note that this is an association study. We were also not able to assess joint functional status following the TKA surgery [20]. Finally, though standard in this literature, the term “use” is applied throughout; our data speak directly to filled opioid prescriptions and not to the directly observed consumption by patients.

## Conclusions

In this high-risk population, chronic opioid use after TKA surgery occurred in more than two-thirds of patients. Preoperative opioids, high opioid dosing during the month following TKA surgery, concomitant benzodiazepines and mood therapies, and comorbid conditions were significant risk factors for chronic use. Longitudinal analysis revealed five distinct opioid use phenotypes, with more than one-third of patients being in the increasing or sustained high opioid use groups. These results demonstrate that sustained and high opioid use following TKA surgery is common; interventional efforts are urgently needed to reduce perioperative opioids with the hopes of mitigating long-term use and abuse.

## Supplementary Information

**Additional file 1:  Table S1.** Diagnostic codes used to develop total knee replacement phenotype. **Table S2.** Demographic and clinical characteristics for total knee replacement trajectory groups, SC Medicaid 2014-2017. **Table S3.** Multinomial model predicting group membership. **Table S4.** Causes of ED visits for group 2 during the outcome period. **Table S5.** Causes of hospitalizations for group 2 during the outcome period. **Table S6.** Causes of ED visits for group 5 during the outcome period. **Table S7.** Causes of hospitalizations for group 5 during the outcome period.

## Data Availability

Will review based on specific requests and data use agreement/consent by local IRB. Data used in this project were received through a contract (A201912450A) with the SC Department of Health and Human Services (SCDHHS) for Drug Utilization Review services. Sharing of data will require a formal request and approval by the above listed agencies.

## Abbreviations

TKA: Total knee arthroplasties
MME: Morphine milligram equivalent
COU: Chronic opioid use
OA: Osteoarthritis of the knee
AAOS: American Academy of Orthopedic Surgeons
NSAIDS: Nonsteroidal antiinflammatory drugs
COX-2: Cyclooxygenase-2
RFA: Revenue and Fiscal Affairs Office
RRs: Risk ratios
GLMM: Generalized linear mixed model
GBTM: Group-based trajectory models
BIC: Bayesian information criterion
PMDI: Periarticular multimodal drug injections
