# Supplementary material for: Risk factors and trajectories of opioid use following total knee replacement
Source: Knee Surg Relat Res. 2022 Apr 5;34:18. doi: 10.1186/s43019-022-00148-0 (PMC8981598; doi:10.1186/s43019-022-00148-0)
Supplement: Supplementary file 1 — Additional file 1: Table S1. Diagnostic codes used to develop total knee replacement phenotype. Table S2. Demographic and clinical characteristics for total knee replacement trajectory groups, SC Medicaid 2014-2017. Table S3. Multinomial model predicting group membership. Table S4. Causes of ED visits for group 2 during the outcome period. Table S5. Causes of hospitalizations for group 2 during the outcome period. Table S6. Causes of ED visits for group 5 during the outcome period. Table S7. Causes of hospitalizations for group 5 during the outcome period. [file 43019_2022_148_MOESM1_ESM.docx]

| **Table S1: Diagnostic codes used to develop total knee replacement phenotype** | | |
| --- | --- | --- |
| **Procedure Codes** | | **Name** |
| **ICD-9** | **ICD-10** |  |
|  | 0SRC069 | Replacement of Right Knee Joint with Oxidized Zirconium on Polyethylene Synthetic Substitute, Cemented, Open Approach |
|  | 0SRC06A | Replacement of Right Knee Joint with Oxidized Zirconium on Polyethylene Synthetic Substitute, Uncemented, Open Approach |
|  | 0SRC06Z | Replacement of Right Knee Joint with Oxidized Zirconium on Polyethylene Synthetic Substitute, Open Approach |
|  | 0SRC07Z | Replacement of Right Knee Joint with Autologous Tissue Substitute, Open Approach |
|  | 0SRC0J9 | Replacement of Right Knee Joint with Synthetic Substitute, Cemented, Open Approach |
|  | 0SRC0JA | Replacement of Right Knee Joint with Synthetic Substitute, Uncemented, Open Approach |
|  | 0SRC0JZ | Replacement of Right Knee Joint with Synthetic Substitute, Open Approach |
|  | 0SRC0KZ | Replacement of Right Knee Joint with Nonautologous Tissue Substitute, Open Approach |
|  | 0SRC0L9 | Replacement of Right Knee Joint with Unicondylar Synthetic Substitute, Cemented, Open Approach |
|  | 0SRC0LA | Replacement of Right Knee Joint with Unicondylar Synthetic Substitute, Uncemented, Open Approach |
|  | 0SRC0LZ | Replacement of Right Knee Joint with Unicondylar Synthetic Substitute, Open Approach |
|  | 0SRD069 | Replacement of Left Knee Joint with Oxidized Zirconium on Polyethylene Synthetic Substitute, Cemented, Open Approach |
|  | 0SRD06A | Replacement of Left Knee Joint with Oxidized Zirconium on Polyethylene Synthetic Substitute, Uncemented, Open Approach |
|  | 0SRD06Z | Replacement of Left Knee Joint with Oxidized Zirconium on Polyethylene Synthetic Substitute, Open Approach |
|  | 0SRD07Z | Replacement of Left Knee Joint with Autologous Tissue Substitute, Open Approach |
|  | 0SRD0J9 | Replacement of Left Knee Joint with Synthetic Substitute, Cemented, Open Approach |
|  | 0SRD0JA | Replacement of Left Knee Joint with Synthetic Substitute, Uncemented, Open Approach |
|  | 0SRD0JZ | Replacement of Left Knee Joint with Synthetic Substitute, Open Approach |
|  | 0SRD0KZ | Replacement of Left Knee Joint with Nonautologous Tissue Substitute, Open Approach |
|  | 0SRD0L9 | Replacement of Left Knee Joint with Unicondylar Synthetic Substitute, Cemented, Open Approach |
|  | 0SRD0LA | Replacement of Left Knee Joint with Unicondylar Synthetic Substitute, Uncemented, Open Approach |
|  | 0SRD0LZ | Replacement of Left Knee Joint with Unicondylar Synthetic Substitute, Open Approach |
|  | 0SRT07Z | Replacement of Right Knee Joint, Femoral Surface with Autologous Tissue Substitute, Open Approach |
|  | 0SRT0J9 | Replacement of Right Knee Joint, Femoral Surface with Synthetic Substitute, Cemented, Open Approach |
|  | 0SRT0JA | Replacement of Right Knee Joint, Femoral Surface with Synthetic Substitute, Uncemented, Open Approach |
|  | 0SRT0JZ | Replacement of Right Knee Joint, Femoral Surface with Synthetic Substitute, Open Approach |
|  | 0SRT0KZ | Replacement of Right Knee Joint, Femoral Surface with Nonautologous Tissue Substitute, Open Approach |
|  | 0SRU07Z | Replacement of Left Knee Joint, Femoral Surface with Autologous Tissue Substitute, Open Approach |
|  | 0SRU0J9 | Replacement of Left Knee Joint, Femoral Surface with Synthetic Substitute, Cemented, Open Approach |
|  | 0SRU0JA | Replacement of Left Knee Joint, Femoral Surface with Synthetic Substitute, Uncemented, Open Approach |
|  | 0SRU0JZ | Replacement of Left Knee Joint, Femoral Surface with Synthetic Substitute, Open Approach |
|  | 0SRU0KZ | Replacement of Left Knee Joint, Femoral Surface with Nonautologous Tissue Substitute, Open Approach |
|  | 0SRV07Z | Replacement of Right Knee Joint, Tibial Surface with Autologous Tissue Substitute, Open Approach |
|  | 0SRV0J9 | Replacement of Right Knee Joint, Tibial Surface with Synthetic Substitute, Cemented, Open Approach |
|  | 0SRV0JA | Replacement of Right Knee Joint, Tibial Surface with Synthetic Substitute, Uncemented, Open Approach |
|  | 0SRV0JZ | Replacement of Right Knee Joint, Tibial Surface with Synthetic Substitute, Open Approach |
|  | 0SRV0KZ | Replacement of Right Knee Joint, Tibial Surface with Nonautologous Tissue Substitute, Open Approach |
|  | 0SRW07Z | Replacement of Left Knee Joint, Tibial Surface with Autologous Tissue Substitute, Open Approach |
|  | 0SRW0J9 | Replacement of Left Knee Joint, Tibial Surface with Synthetic Substitute, Cemented, Open Approach |
|  | 0SRW0JA | Replacement of Left Knee Joint, Tibial Surface with Synthetic Substitute, Uncemented, Open Approach |
|  | 0SRW0JZ | Replacement of Left Knee Joint, Tibial Surface with Synthetic Substitute, Open Approach |
|  | 0SRW0KZ | Replacement of Left Knee Joint, Tibial Surface with Nonautologous Tissue Substitute, Open Approach |
| 8154 |  | Total knee replacement |

| **Table S2: Demographic and clinical characteristics for total knee replacement trajectory groups, SC Medicaid 2014-2017** | | | | | | | | |
| --- | --- | --- | --- | --- | --- | --- | --- | --- |
| **Measure^a^** | **Level** | **TRAJECTORY GROUP** | | | | | **Total** | **p-value** |
|  |  | **Group 1**  **Little or**  **no use** | **Group 2**  **Increasing**  **use** | **Group 3**  **Rapid opioid**  **wean** | **Group 4:**  **Slow opioid**  **wean** | **Group 5:**  **Sustained**  **high use** |  |  |
| Unique patients^b^ |  | 428 | 181 | 399 | 198 | 388 | 1507 |  |
| Number of total knee surgeries | total surgeries | 451(27.3%) | 188 (11.3%) | 409 (24.4%) | 203 (12.1%) | 415 (24.9%) | 1666 | 0.087 |
|  | first surgery | 389 (26.2%) | 170 (11.4%) | 370 (24.9%) | 177 (11.9%) | 380 (25.6%) | 1486 |  |
|  | second surgery | 62 (34.4%) | 18 (10.0%) | 39 (21.7%) | 26 (14.4%) | 35 (19.4%) | 180 |  |
| Surgeries with chronic outcome |  | 100 (22.2%) | 188 (100%) | 243 (59.4%) | 203 (100%) | 415 (100%) | 1149 (69.0%) | <0.0001 |
| Age | mean (std) | 62.7 (10.4) | 59.9 (9.9) | 60.2 (9.9) | 57.1 (9.3) | 57.2 (9.2) | 59.7 (10) | <0.0001 |
| Age category N(%) | 18-34 years | 2 (0.4%) | 2 (1.1%) | 3 (0.7%) | 1 (0.5%) | 5 (1.2%) | 13 (0.8%) | <0.0001 |
|  | 35-44 years | 16 (3.5%) | 12 (6.4%) | 19 (4.6%) | 18 (8.9%) | 24 (5.8%) | 89 (5.3%) |  |
|  | 45-54 years | 74 (16.4%) | 29 (15.4%) | 90 (22.0%) | 57 (28.1%) | 129 (31.1%) | 379 (22.7%) |  |
|  | 55+ years | 359 (79.6%) | 145 (77.1%) | 297 (72.6%) | 127 (62.6%) | 257 (61.9%) | 1185 (71.1%) |  |
| Sex | % male | 86 (19.1%) | 36 (19.2%) | 73 (17.9%) | 43 (21.2) | 93 (22.4) | 331 (19.9%) | 0.52 |
| Race/ethnicity | White | 141 (31.3%) | 58 (30.9%) | 130 (31.8%) | 75 (36.9%) | 192 (46.3%) | 596 (35.8%) | <0.0001 |
|  | Black | 203 (45.0%) | 88 (46.8%) | 211 (51.6%) | 86 (42.4%) | 152 (36.6%) | 740 (44.4%) |  |
|  | Hispanic | 8 (1.8%) | 2 (1.1%) | 3 (0.7%) | 1 (0.5%) | 1 (0.2%) | 15 (0.9%) |  |
|  | Unknown/Other | 99 (22.0%) | 40 (21.3%) | 65 (15.9%) | 41 (20.2%) | 70 (16.9%) | 315 (18.9%) |  |
| **Baseline measures (pre-surgical period^c^)** | | | | | | | | |
| Opioid naïve^d^ | 0 days | 268 (59.4%) | 50 (26.6%) | 167 (40.8%) | 29 (14.3%) | 16 (3.9%) | 530 (31.8%) | <0.0001 |
| Opioid use during pre-surgical period | 0-49 MME/day | 449 (99.6%) | 187 (99.5%) | 400 (97.8%) | 188 (92.6%) | 284 (68.4%) | 1508 (90.5%) | <0.0001 |
|  | 50-89 MME/day | 2 (0.4%) | 1 (0.5%) | 9 (2.2%) | 11 (5.4%) | 97 (23.4%) | 120 (7.2%) |  |
|  | ≥90MME/day | 0 (0.0%) | 0 (0.0%) | 0 (0.0%) | 4 (2.0%) | 34 (8.2%) | 38 (2.3%) |  |
| **Opioid use^e^ among all patients** | | | | | | | | |
| Mean opioid days [mean(std)] | pre-surgical period | 11.7 (21.7) | 34.8 (31.6) | 19.9 (26.4) | 44.4 (33.9) | 67.8 (28) | 34.3 (35) | <0.0001 |
|  | exposure period^f^ | 8.4 (8.7) | 15.9 (9.4) | 16.8 (8.6) | 22.1 (9) | 24.8 (6.9) | 17.1 (10.4) | <0.0001 |
|  | ≥90 days post-surgery | 4.9 (15.2) | 76.7 (48.8) | 13 (21.4) | 60.7 (39.5) | 154.1 (31.1) | 59 (66.9) | <0.0001 |
| Mean MME [mean(std)] | pre-surgical period | 4.3 (9.1) | 10.2 (12.1) | 6.4 (12.3) | 18.9 (22.6) | 42.6 (43) | 17.6 (29.6) | <0.0001 |
|  | exposure period | 19.7 (23.2) | 35.6 (26.7) | 44 (33.8) | 65.8 (50.9) | 84 (69.6) | 49.1 (51.2) | <0.0001 |
|  | ≥90 days post-surgery | 0.4 (0.8) | 12.6 (7.6) | 1.2 (1.7) | 11.2 (7.1) | 56.8 (68.8) | 18.5 (42.7) | <0.0001 |
| Percent with opioid use | pre-surgical period | 183 (40.6%) | 138 (73.4%) | 242 (59.2%) | 174 (85.7%) | 399 (96.1%) | 1136 (68.2%) | <0.0001 |
|  | exposure period | 277 (61.4%) | 171 (91.0%) | 386 (94.4%) | 194 (95.6%) | 412 (99.3%) | 1440 (86.4%) | <0.0001 |
|  | ≥ 90 days post-surgery | 100 (22.2%) | 188 (100%) | 242 (59.2%) | 203 (100%) | 415 (100%) | 1148 (68.9%) | <0.0001 |
| **Opioid prescriptions dispensed during exposure period** | | | | | | | | |
| Mean opioid days | mean(std) | 13.7 (7.2) | 17.5 (8.4) | 17.7 (7.8) | 23 (7.9) | 25 (6.5) | 19.7 (8.5) | <0.0001 |
| Opioid type | long acting | 2 (0.4%) | 3 (1.6%) | 0 (0.0%) | 1 (0.5%) | 1 (0.2%) | 7 (0.4%) | <0.0001 |
|  | short acting | 258 (57.2%) | 156 (83.0%) | 349 (85.3%) | 157 (77.3%) | 335 (80.7%) | 1255 (75.3%) |  |
|  | both | 20 (4.4%) | 13 (6.9%) | 37 (9.0%) | 35 (17.2%) | 77 (18.6%) | 182 (10.9%) |  |
|  | none (no opioids prescribed) | 171 (37.9%) | 16 (8.5%) | 23 (5.6%) | 10 (4.9%) | 2 (0.5%) | 222 (13.3%) |  |
| Single vs combination opioid | single | 90 (20.0%) | 34 (18.1%) | 73 (17.8%) | 37 (18.2%) | 67 (16.1%) | 301 (18.1%) | <0.0001 |
|  | combination | 123 (27.3%) | 86 (45.7%) | 174 (42.5%) | 76 (37.4%) | 146 (35.2%) | 605 (36.3%) |  |
|  | both | 67 (14.9%) | 52 (27.7%) | 139 (34.0%) | 80 (39.4%) | 200 (48.2%) | 538 (32.3%) |  |
|  | none (no opioids prescribed) | 171 (37.9%) | 16 (8.5%) | 23 (5.6%) | 10 (4.9%) | 2 (0.5%) | 222 (13.3%) |  |
| MME/day | mean(std) over 30 days | 32.1 (21.9) | 39.1 (25.4) | 46.7 (33) | 69.2 (49.9) | 84.6 (69.5) | 56.9 (51) | <0.0001 |
| Opioid prescription count | mean (std) | 1.2 (1.2) | 2.2 (1.4) | 2.5 (1.4) | 3.2 (1.7) | 3.6 (1.6) | 2.5 (1.7) | <0.0001 |
| Provider practice specialty | surgeon | 205 (45.5%) | 114 (60.6%) | 298 (72.9%) | 156 (76.8%) | 270 (65.1%) | 1043 (62.6%) | <0.0001 |
|  | other | 24 (5.3%) | 13 (6.9%) | 35 (8.6%) | 18 (8.9%) | 43 (10.4%) | 133 (8.0%) |  |
|  | primary care provider | 42 (9.3%) | 38 (20.2%) | 46 (11.2%) | 14 (6.9%) | 83 (20.0%) | 223 (13.4%) |  |
|  | missing | 9 (1.5%) | 7 (3.8%) | 7 (1.7%) | 5 (2.5%) | 17 (4%) | 45 (2.7%) |  |
|  | none (no opioids prescribed) | 171 (37.9%) | 16 (8.5%) | 23 (5.6%) | 10 (4.9%) | 2 (0.5%) | 222 (13.3%) |  |
| **Concomitant medications dispensed during exposure period (all patients)** | | | | | | | | |
|  | NSAIDS, APAP | 87 (19.3%) | 46 (24.5%) | 87 (21.3%) | 44 (21.7%) | 98 (23.6%) | 362 (21.7%) | 0.50 |
|  | Antidepressants | 54 (12.0%) | 41 (21.8%) | 84 (20.5%) | 47 (23.2%) | 133 (32.0%) | 359 (21.5%) | <0.0001 |
|  | Antipsychotics | 18 (4.0%) | 8 (4.3%) | 16 (3.9%) | 19 (9.4%) | 35 (8.4%) | 96 (5.8%) | 0.0042 |
|  | Gabapentin | 31 (6.9%) | 22 (11.7%) | 60 (14.7%) | 40 (19.7%) | 74 (17.8%) | 227 (13.6%) | <0.0001 |
|  | Pregabalin | 6 (1.3%) | 10 (5.3%) | 9 (2.2%) | 4 (2.0%) | 24 (5.8%) | 53 (3.2%) | 0.0004 |
|  | Benzodiazepines | 20 (4.4%) | 25 (13.3%) | 45 (11.0%) | 42 (20.7%) | 126 (30.4%) | 258 (15.5%) | <0.0001 |
|  | Selected sedatives/hypnotics^g^ | 18 (4.0%) | 12 (6.4%) | 28 (6.8%) | 19 (9.4%) | 53 (12.8%) | 130 (7.8%) | <0.0001 |
|  | Muscle relaxants | 18 (4.0%) | 14 (7.4%) | 36 (8.8%) | 31 (15.3%) | 87 (21.0%) | 186 (11.2%) | <0.0001 |
|  | Duloxetine | 9 (2.0%) | 8 (4.3%) | 19 (4.6%) | 14 (6.9%) | 31 (7.5%) | 81 (4.9%) | 0.0013 |
| **Alternative pain treatments** | | | | | | | | |
|  | Physical therapy | 76 (16.9%) | 56 (29.8%) | 78 (19.1%) | 35 (17.2%) | 61 (14.7%) | 306 (18.4%) | 0.0017 |
|  | Occupational therapy | 7 (1.6%) | 5 (2.7%) | 12 (2.9%) | 1 (0.5%) | 7 (1.7%) | 32 (1.9%) | 0.28 |
|  | Acupuncture | 0 | 0 | 0 | 0 | 0 | 0 | NA |
| **Surgery and process of care variables** | | | | | | | | |
| Rehospitalization N(%) | within 30 days of discharge | 5 (1.1%) | 3 (1.6%) | 4 (1.0%) | 3 (1.5%) | 11 (2.7%) | 26 (1.6%) | 0.35 |
| Skilled nursing and Rehab facilities |  | 33 (7.3%) | 10 (5.3%) | 28 (6.8%) | 9 (4.4%) | 22 (5.3%) | 102 (6.1%) | 0.56 |
| Days to post-discharge surgeon visit | No visits | 384 (85.1%) | 137 (72.9%) | 325 (79.5%) | 164 (80.8%) | 316 (76.1%) | 1326 (79.6%) | 0.013 |
|  | 14 days or less | 43 (9.5%) | 31 (16.5%) | 51 (12.5%) | 28 (13.8%) | 58 (14.0%) | 211 (12.7%) |  |
|  | More than 14 days | 24 (5.3%) | 20 (10.6%) | 33 (8.1%) | 11 (5.4%) | 41 (9.9%) | 129 (7.7%) |  |
| Days to first PCP visit | No visits | 300 (66.5%) | 131 (69.7%) | 282 (68.9%) | 137 (67.5%) | 260 (62.7%) | 1110 (66.6%) | 0.56 |
|  | 14 days or less | 79 (17.5%) | 31 (16.5%) | 66 (16.1%) | 37 (18.2%) | 92 (22.2%) | 305 (18.3%) |  |
|  | More than 14 days | 72 (16.0%) | 26 (13.8%) | 61 (14.9%) | 29 (14.3%) | 63 (15.2%) | 251 (15.1%) |  |
| Total visits during exposure period [mean(std)] | Primary care provider | 0.9 (1.9) | 0.9 (2.2) | 0.8 (1.8) | 0.8 (2.2) | 1.1 (2.4) | 0.9 (2.1) | 0.4057 |
|  | Surgeon | 0.2 (0.6) | 0.3 (0.6) | 0.3 (0.5) | 0.3 (0.7) | 0.3 (0.7) | 0.3 (0.6) | 0.0289 |
|  | Other prescribers | 0.2 (0.8) | 0.3 (0.9) | 0.3 (1) | 0.4 (0.8) | 0.4 (1.2) | 0.3 (1) | 0.0862 |
|  | Non-prescribers^h^ | 0.2 (1.1) | 0.2 (1.2) | 0.2 (1.3) | 0 (0.2) | 0.2 (1.1) | 0.2 (1.1) | <0.0001 |
|  | total visits | 1.5 (2.5) | 1.7 (2.9) | 1.6 (2.9) | 1.4 (2.3) | 2 (3.3) | 1.7 (2.8) | 0.0851 |
| Hospital length of stay | 4 or more nights | 363 (80.5%) | 149 (79.3%) | 331 (80.9%) | 164 (80.8%) | 308 (74.2%) | 1315 (78.9%) | 0.12 |
|  | 3 nights or less | 88 (19.5%) | 39 (20.7%) | 78 (19.1%) | 39 (19.2%) | 107 (25.8%) | 351 (21.1%) |  |
| Count of unique prescribers (pharmacy data) | | 0.9 (0.8) | 1.5 (0.8) | 1.5 (0.8) | 1.8 (0.9) | 2 (0.8) | 1.5 (0.9) | <0.0001 |
| Number of unique non-prescribers visited mean(std) | | 0.1 (0.3) | 0.1 (0.3) | 0.1 (0.4) | 0 (0.2) | 0.1 (0.4) | 0.1 (0.3) | 0.0283 |
| APAP, acetaminophen; MME, morphine milligram equivalent; NSAIDS, nonsteroidal anti-inflammatory drugs; PCP, primary care provider. | | | | | | | | |
| 1. Because some patients had multiple surgeries, unless otherwise indicated, all measures are aggregated by surgical procedures rather than by individual patients. 2. Sum of unique patients does not reflect value in total column because some patients with multiple surgeries may appear in different trajectory groups. 3. The pre-surgical period was defined as the 90 days prior to procedure 4. Opioid naïve for analyses defined as 0 opioid days during the pre-surgical period 5. Opioid use was assessed by dispensed opioid prescriptions 6. The exposure period was defined as the 30 days immediately after discharge from procedure 7. This category includes non-benzodiazepine sedative/hypnotics and selected anxiolytics 8. Includes other health professions who can submit Medicaid claims, such as therapists | | | | | | | | |

| **Table S3: Multinomial model predicting group membership** | | | | | | | | |
| --- | --- | --- | --- | --- | --- | --- | --- | --- |
| **(reference = GROUP 1)** | | **MME Exposure model** | | | | | | |
| **Variable** | **Level** | **Odds Ratios (95% CI)** | | | | | | |
|  |  | **Group 2** | | **Group 3** | | **Group 4** | | **Group 5** |
| Opioid naïve^a^ | No vs. Yes | 1.71 (1.39, 2.10) | 1.18 (1.01, 1.38) | | 2.21 (1.73, 2.81) | | 4.34 (3.21, 5.87) | |
| Mean daily MME during exposure period^b^ | 0<MME<50 vs. 0 MME | 1.13 (0.74, 1.72) | 0.98 (0.69, 1.38) | | 0.58 (0.38, 0.86) | | 0.49 (0.31, 0.78) | |
|  | 50≤ MME<90 vs. 0 MME | 1.19 (0.71, 2.00) | 1.11 (0.72, 1.70) | | 1.09 (0.67, 1.76) | | 1.97 (1.18, 3.30) | |
|  | MME≥90 vs. 0 MME | 1.49 (0.53, 4.23) | 3.36 (1.44, 7.82) | | 4.38 (1.79, 10.7) | | 10.6 (4.31, 25.9) | |
| Hospital length of stay | 4+ days vs. 3 or fewer days | 1.09 (0.86, 1.39) | 1.11 (0.91, 1.35) | | 1.19 (0.92, 1.53) | | 1.51 (1.20, 1.90) | |
| Number of unique prescribers during exposure period | per additional prescriber | 0.93 (0.63, 1.37) | 0.80 (0.58, 1.11) | | 0.84 (0.58, 1.20) | | 0.99 (0.70, 1.40) | |
| Patient age | per additional year | 1.00 (0.97, 1.02) | 1.00 (0.98, 1.02) | | 0.98 (0.96, 1.00) | | 0.99 (0.97, 1.02) | |
| Number of opioid prescriptions during exposure period | per additional prescription | 1.51 (1.15, 1.97) | 1.78 (1.42, 2.22) | | 2.01 (1.57, 2.57) | | 1.85 (1.46, 2.34) | |
| Race-ethnicity | White | ref | ref | | ref | | ref | |
|  | Minority race-ethnicity | 1.15 (0.92, 1.44) | 1.05 (0.88, 1.26) | | 1.05 (0.84, 1.32) | | 0.99 (0.80, 1.22) | |
| Sex | Male vs. female | 1.05 (0.81, 1.37) | 1.18 (0.95, 1.47) | | 1.09 (0.83, 1.42) | | 0.94 (0.73, 1.21) | |
| Concomitant medications dispensed during exposure period | NSAIDS, APAP | 0.96 (0.76, 1.20) | 0.86 (0.71, 1.04) | | 0.78 (0.61, 0.99) | | 0.82 (0.66, 1.03) | |
|  | Antidepressants | 1.22 (0.93, 1.59) | 1.25 (1.00, 1.57) | | 1.15 (0.87, 1.52) | | 1.30 (1.01, 1.68) | |
|  | Antipsychotics | 0.95 (0.58, 1.57) | 0.89 (0.59, 1.35) | | 1.60 (1.02, 2.51) | | 1.33 (0.86, 2.05) | |
|  | Gabapentin | 1.00 (0.73, 1.39) | 1.27 (0.98, 1.64) | | 1.39 (1.03, 1.88) | | 1.13 (0.85, 1.51) | |
|  | Pregabalin | 1.46 (0.81, 2.62) | 0.91 (0.51, 1.63) | | 0.80 (0.38, 1.66) | | 0.97 (0.53, 1.76) | |
|  | Benzodiazepines | 1.38 (0.97, 1.95) | 1.21 (0.89, 1.65) | | 1.44 (1.02, 2.03) | | 1.49 (1.08, 2.04) | |
|  | Selected sedatives/hypnotics^c^ | 0.99 (0.65, 1.50) | 1.01 (0.72, 1.42) | | 1.06 (0.71, 1.58) | | 1.15 (0.80, 1.64) | |
|  | Muscle relaxants | 1.04 (0.70, 1.54) | 1.12 (0.81, 1.55) | | 1.37 (0.96, 1.96) | | 1.50 (1.07, 2.10) | |
|  | Duloxetine | 1.17 (0.69, 2.00) | 1.42 (0.92, 2.19) | | 1.66 (1.01, 2.73) | | 1.38 (0.86, 2.22) | |
| Medical comorbidities | Congestive heart failure | 0.75 (0.56, 1.01) | 0.98 (0.78, 1.23) | | 0.76 (0.56, 1.04) | | 0.97 (0.74, 1.27) | |
|  | Valvular disease | 0.79 (0.59, 1.06) | 1.00 (0.80, 1.25) | | 0.86 (0.64, 1.17) | | 1.04 (0.80, 1.36) | |
|  | Pulmonary circulation disease | 1.15 (0.79, 1.67) | 0.84 (0.60, 1.18) | | 1.49 (1.03, 2.17) | | 1.03 (0.71, 1.51) | |
|  | Peripheral vascular disease | 1.47 (1.12, 1.93) | 1.18 (0.93, 1.49) | | 1.18 (0.87, 1.60) | | 1.21 (0.92, 1.60) | |
|  | Hypertension | 1.10 (0.79, 1.52) | 1.09 (0.84, 1.41) | | 1.06 (0.77, 1.46) | | 1.03 (0.76, 1.40) | |
|  | Hypertension with complications | 0.95 (0.70, 1.30) | 0.98 (0.76, 1.26) | | 1.09 (0.78, 1.51) | | 1.05 (0.78, 1.41) | |
|  | Paralysis | 1.38 (0.82, 2.31) | 1.37 (0.87, 2.16) | | 1.54 (0.89, 2.64) | | 1.24 (0.73, 2.13) | |
|  | Other neurological disorders | 1.07 (0.83, 1.38) | 1.00 (0.81, 1.25) | | 0.89 (0.68, 1.18) | | 0.95 (0.74, 1.21) | |
|  | Chronic pulmonary disease | 1.06 (0.87, 1.29) | 0.97 (0.83, 1.15) | | 1.10 (0.89, 1.35) | | 1.15 (0.95, 1.40) | |
|  | Diabetes without chronic complications | 0.95 (0.78, 1.17) | 1.08 (0.92, 1.28) | | 1.03 (0.83, 1.29) | | 0.90 (0.74, 1.11) | |
|  | Diabetes with chronic complications | 1.06 (0.84, 1.33) | 0.98 (0.80, 1.18) | | 0.88 (0.68, 1.15) | | 1.21 (0.96, 1.52) | |
|  | Hypothyroidism | 1.12 (0.88, 1.42) | 1.12 (0.91, 1.36) | | 1.00 (0.76, 1.30) | | 1.14 (0.90, 1.45) | |
|  | Renal failure | 1.22 (0.92, 1.63) | 1.04 (0.82, 1.32) | | 0.98 (0.70, 1.36) | | 1.09 (0.82, 1.45) | |
|  | Liver disease | 1.29 (0.94, 1.77) | 1.07 (0.81, 1.42) | | 1.23 (0.89, 1.71) | | 1.03 (0.76, 1.41) | |
|  | Peptic ulcer Disease | 0.47 (0.21, 1.06) | 0.77 (0.47, 1.27) | | 0.51 (0.24, 1.06) | | 1.04 (0.61, 1.77) | |
|  | Rheumatoid arthritis | 1.10 (0.86, 1.39) | 0.99 (0.81, 1.21) | | 0.82 (0.63, 1.07) | | 1.22 (0.97, 1.54) | |
|  | Coagulopathy | 0.85 (0.52, 1.38) | 1.04 (0.73, 1.50) | | 0.76 (0.45, 1.30) | | 0.94 (0.60, 1.46) | |
|  | Obesity | 1.06 (0.86, 1.30) | 1.03 (0.87, 1.21) | | 0.96 (0.78, 1.19) | | 0.99 (0.81, 1.22) | |
|  | Weight loss | 0.80 (0.54, 1.19) | 1.07 (0.79, 1.45) | | 0.94 (0.64, 1.38) | | 0.83 (0.57, 1.19) | |
|  | Fluid and electrolyte disorders | 1.07 (0.87, 1.33) | 0.88 (0.73, 1.05) | | 1.03 (0.82, 1.28) | | 1.07 (0.87, 1.31) | |
|  | Chronic blood loss anemia | 2.03 (1.31, 3.16) | 1.65 (1.12, 2.44) | | 1.97 (1.21, 3.20) | | 1.17 (0.70, 1.96) | |
|  | Deficiency Anemias | 0.83 (0.68, 1.03) | 0.92 (0.78, 1.09) | | 0.85 (0.68, 1.06) | | 0.88 (0.72, 1.08) | |
|  | Alcohol abuse | 0.93 (0.58, 1.50) | 1.24 (0.84, 1.83) | | 1.20 (0.76, 1.89) | | 1.16 (0.76, 1.76) | |
|  | Substance use disorders | 1.53 (1.08, 2.16) | 1.42 (1.04, 1.93) | | 1.38 (0.97, 1.95) | | 1.57 (1.14, 2.17) | |
|  | Psychoses | 0.85 (0.65, 1.12) | 0.92 (0.74, 1.16) | | 0.69 (0.51, 0.92) | | 0.90 (0.69, 1.16) | |
|  | Depression | 1.12 (0.90, 1.40) | 0.91 (0.75, 1.09) | | 1.04 (0.82, 1.31) | | 1.13 (0.91, 1.40) | |
| APAP, acetaminophen; MME, morphine milligram equivalent; NSAIDS, nonsteroidal anti-inflammatory drugs; 95% CI, 95% confidence interval. | | | | | | | | |
| 1. Opioid naïve for analyses defined as 0 opioid days during the pre-surgical period 2. The exposure period was defined as the 30 days immediately after discharge from procedure 3. This category includes non-benzodiazepine sedative/hypnotics and selected anxiolytics | | | | | | | | |

| Table S4: Causes of ED Visits for Group 2 During the Outcome Period | |  |  |  |
| --- | --- | --- | --- | --- |
| **Cause of ED Visit Based on Primary ICD 9/10 Code** | **Count** | **Mean Opioid MME (mg/day) Month Prior to ED Visit** | **Mean Opioid MME (mg/day) Month During ED Visit** | **Mean Opioid MME (mg/day) Month After ED Visit** |
| Cardiovascular | 5 | 3.5 | 3.7 | 5.7 |
| Infection | 10 | 11.8 | 17.6 | 11.4 |
| Acute Injury | 9 | 8.9 | 16.3 | 14.8 |
| Joint, Bone, or Muscular Issue | 9 | 13.5 | 16.9 | 8.7 |
| Substance Use Disorder | 1 | 0.0 | 0.0 | 18.7 |
| Comorbid Condition or Miscellaneous | 43 | 13.7 | 15.3 | 11.2 |
|  |  |  |  |  |

| Table S5: Causes of Hospitalizations for Group 2 During the Outcome Period | |  |  |  |
| --- | --- | --- | --- | --- |
| **Cause of Hospitalization Based on Primary ICD 9/10 Code** | **Count** | **Mean Opioid MME (mg/day) Month Prior to Hospitalization** | **Mean Opioid MME (mg/day) Month During Hospitalization** | **Mean Opioid MME (mg/day) Month After Hospitalization** |
| Cardiovascular | 3 | 0.7 | 0.0 | 5.3 |
| Infection | 4 | 25.7 | 34.5 | 16.9 |
| Joint, Bone, or Muscular Issue | 2 | 0.0 | 39.2 | 7.3 |
| TKA Surgery Complication | 3 | 12.4 | 18.8 | 6.3 |
| Comorbid Condition or Miscellaneous | 6 | 46.9 | 7.4 | 13.1 |
|  |  |  |  |  |

| Table S6: Causes of ED Visits for Group 5 During the Outcome Period | |  |  |  |
| --- | --- | --- | --- | --- |
| **Cause of ED Visit Based on Primary ICD 9/10 Code** | **Count** | **Mean Opioid MME (mg/day) Month Prior to ED Visit** | **Mean Opioid MME (mg/day) Month During ED Visit** | **Mean Opioid MME (mg/day) Month After ED Visit** |
| Cardiovascular | 7 | 50.6 | 44.6 | 39.3 |
| Infection | 17 | 53.1 | 64.7 | 55.7 |
| Acute Injury | 12 | 68.7 | 79.6 | 75.4 |
| Joint, Bone, or Muscular Issue | 11 | 79.8 | 84.0 | 78.7 |
| Substance Use Disorder | 5 | 24.9 | 49.3 | 35.2 |
| Comorbid Condition or Miscellaneous | 60 | 58.1 | 56.4 | 50.5 |
|  |  |  |  |  |

| Table S7: Causes of Hospitalizations for Group 5 During the Outcome Period | |  |  |  |
| --- | --- | --- | --- | --- |
| **Cause of Hospitalization Based on Primary ICD 9/10 Code** | **Count** | **Mean Opioid MME (mg/day) Month Prior to Hospitalization** | **Mean Opioid MME (mg/day) Month During Hospitalization** | **Mean Opioid MME (mg/day) Month After Hospitalization** |
| Cardiovascular | 2 | 35.3 | 38.7 | 36.7 |
| Infection | 5 | 58.1 | 49.7 | 57.6 |
| Joint, Bone, or Muscular Issue | 3 | 64.5 | 71.5 | 66.0 |
| Acute Injury | 1 | 133.8 | 45.6 | 95.5 |
| TKA Surgery Complication | 2 | 42.5 | 15.7 | 47.0 |
| Comorbid Condition or Miscellaneous | 15 | 68.0 | 69.0 | 56.0 |
